# Modeling of scour hole characteristics under turbulent wall jets using machine learning

**DOI:** 10.1038/s41598-024-66291-8

**Published:** 2024-07-06

**Authors:** Jnana Ranjan Khuntia, Kamalini Devi, Mohd Aamir Mumtaz

**Affiliations:** 1 Civil Engineering Department, Chaitanya Bharathi Institute of Technology (Autonomous), Hyderabad, 500075 India; 2 Civil Engineering Department, College of Engineering, Imam Mohammad Ibn Saud Islamic University (IMSIU), 11432 Riyadh, Saudi Arabia

**Keywords:** Scour depth, Turbulent wall Jets, Multi regression analysis, ANN-PSO, GEP, Civil engineering, Scientific data, Fluid dynamics

## Abstract

The novelty of the present study is to investigate the parameters that depict the scour hole characteristics caused by turbulent wall jets and develop new mathematical relationships for them. Four significant parameters i.e., depth of scouring, location of scour depth, height of the dune and location of dune crest are identified to represent a complete phenomenon of scour hole formation. From the gamma test, densimetric Froude number, apron length, tailwater level, and median sediment size are found to be the key parameters that affect these four dependent parameters. Utilizing the previous data sets, Multi Regression Analysis (linear and non-linear) has been performed to establish the relationships between the dependent parameters and influencing independent parameters. Further, artificial neural network-particle swarm optimisation (ANN-PSO) and gene expression programming (GEP) based models are developed using the available data. In addition, results obtained from these models are compared with proposed regression equations and the best models are identified employing statistical performance parameters. The performance of the ANN-PSO model (RMSE = 1.512, R^2^ = 0.605), (RMSE = 6.644, R^2^ = 0.681), (RMSE = 6.386, R^2^ = 0.727) and (RMSE = 1.754, R^2^ = 0.636) for predicting four significant parameters are more satisfactory than that of regression and other soft computing techniques. Overall, by analysing all the statistical parameters, uncertainty analysis and reliability index, ANN-PSO model shows good accuracy and predicts well as compared to other presented models.

## Introduction

The hydrodynamic function of flowing water entails the removal of sediments from the riverbed or channel, a process commonly referred to as scouring. Out of all the varieties of scouring mechanisms, scour hole formed due to turbulent wall jets occurs when a high-velocity fluid jet impinges on a boundary wall or a submerged structure leading to change in the flow characteristics. Scour around hydraulic structures exposes their foundations, thereby endangering the integrity of the structure, as these exposed foundations are susceptible to damage and failure. Wall jets, characterized by a high breadth-to-depth ratio, develop when flow passes through a sluice opening or downstream of a slab culvert. Typically, a rigid apron is constructed immediately downstream of the sluice gate, over which the jet travels before reaching the erodible sediment bed, initiating the erosion process. In some instances, a launching apron is installed as a protective measure against scour. A scour hole forms adjacent to the rigid apron, followed by ridge development as the jet's scouring capacity diminishes with distance from the sluice gate. The scouring mechanism can be well-characterised by the depth of scouring, location of scour depth, height of the dune and location of dune crest. The structure of the entire scour hole region depends invariably on flow, geometry and sediment characteristics such as the velocity of the jet and discharge, geometry of the channel or water body and bed material composition. Moreover, scour hole characteristics are also influenced by the duration of the jet and the interaction between the jet and the bed. To predict the scour depth, numerous researchers and engineers developed empirical relationships using experimental data and focused on understanding the complex dynamics. Melville and Sutherland^1^ developed a design method for local scour at bridge piers. Melville and Coleman^2^ explored the influence of wall jets on the scour developed near the pier and derived equations by taking the jet momentum coefficient and angle of impingement into account. Ettema et al.^3^, and Simarro et al.^4^, have provided empirical relationships and design considerations for engineers to predict scour depth under turbulent wall jet scenarios. Dey et al.^5^ proposed equations suitable for scouring phenomenon due to impinging wall jets. Maximum scour hole depth at downstream of a sluice gate was investigated by Azamathulla^6^ and Karbasi and Azamathulla^7^ through five soft-computing techniques: artificial neural networks, support vector regression, gene expression programming, grouping method of data handling (GMDH) neural network and adaptive-network-based fuzzy inference system. Considering the issue of scour under wall jets, which reflects a threat to the foundations of hydraulic structures, Aamir and Ahmad^8^ proposed an empirical equation for prediction of scour at downstream of a rigid apron under wall jets. Chen et al.^9^ conducted comprehensive laboratory experiments and also employed computational fluid dynamics (CFD) modeling to simulate turbulent wall jets induced scour characteristics. Li et al.^10^ employed a neural network-based approach to calculate scour depth under wall jet phenomenon, considering flow and sediment parameters as inputs. Scour characteristics at the downstream of stilling basins were studied by Farhoudi et al.^11^ by employing a neuro-fuzzy model. Ebtehaj et al.^12^ used the self-adaptive extreme learning machine technique to predict the equilibrium scour depth around bridge piers. Scour depth at seawalls were investigated by Pourzangbar et al.^13^ through genetic programming (GP) and ANN. The recent studies revealed that the soft-computing models exhibit greater accuracy as compared to empirical equations. Scour depth near spur dikes is studied by Pandey et al.^14^ using two innovative tree-based ensemble models such as stacked boosting regression tree (SBRT) and stacked bagging regression tree (SBGT). Further, three robust AI-based techniques were introduced by Pandey et al.^15^, such as gradient boosting decision tree (GBDT), cascaded forward neural network (CFNN), and kernel ridge regression (KRR), to predict the scour depth around a spur dike in cohesive sediment mixtures.

All the experimental studies may involve physical scale models or field measurements to investigate scour phenomena. They help to establish empirical relationships between the influencing parameters and the magnitude of scour depth. The optimal aim of these studies was to estimate the maximum scour depth however, the understanding of the scour profile and its spatial distribution are not investigated in depth. According to author’s knowledge, so many works are carried out in the study of scour depth modelling but none of them has focused particularly on modelling of four parameters which can completely define the scour hole development in both vertical and horizontal dimensions. It must be noted that predicting the scour hole profile caused by turbulent wall jets is a complex task and relies on accurate flow conditions and sediment properties.

**Figure 1:**
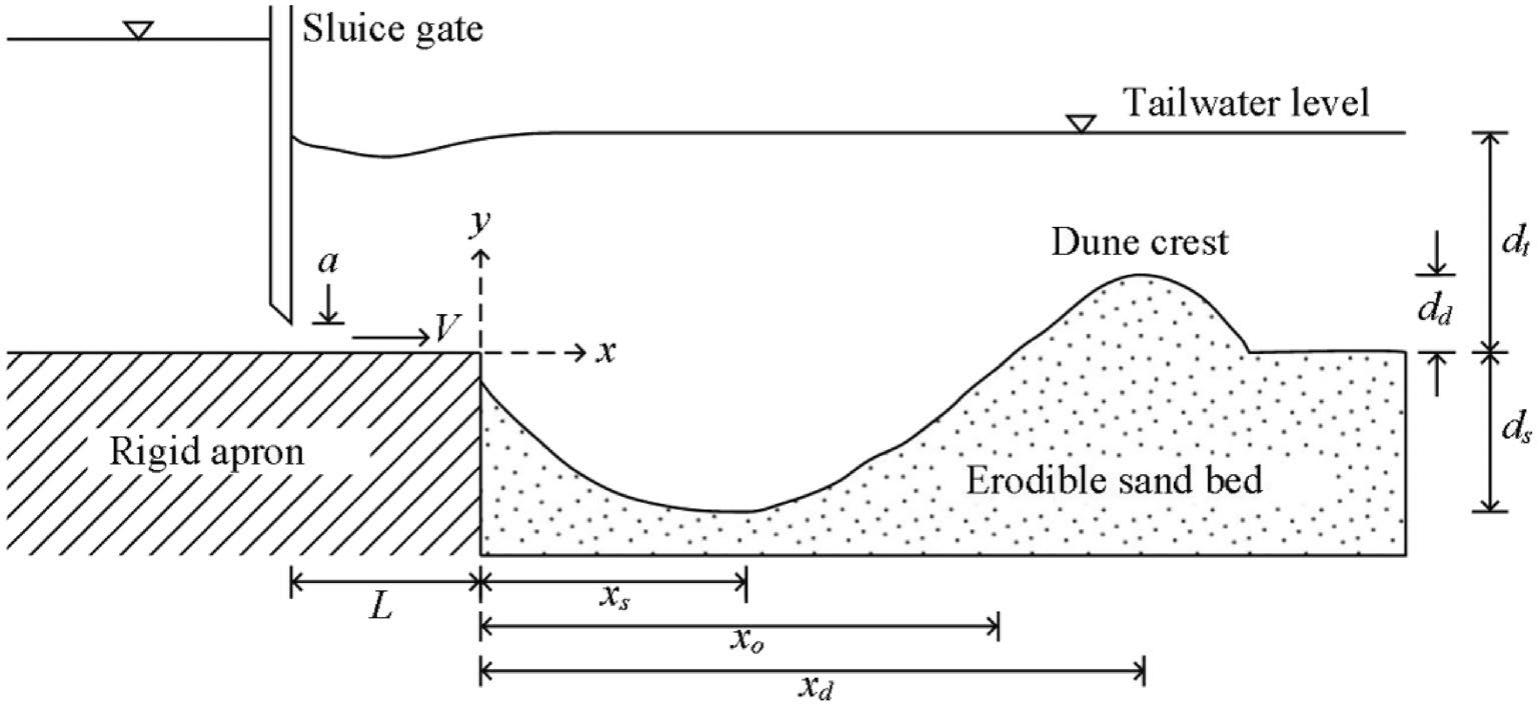
Definition sketch of developed scour hole under wall jet.

The prediction of the scour hole characteristics involves the calculation of scour depth, location of scour depth, depth of the dune and location of dune crest, as shown in Fig. 1. Complete information on these four parameters can probably render a detailed knowledge of the specific scouring mechanism and its form. As the recent studies have focused only on the multidisciplinary nature of scour depth prediction through experimental, numerical, and machine learning approaches, models for other three parameters (location of scour depth, height of the dune and location of dune crest) are further required to be developed to showcase the complete phenomenon of scour characteristics under turbulent wall jet conditions.

Aamir and Ahmad^8^ have provided a wide range of data sets through extensive experimentation in a rigid apron under wall jets. They proposed empirical equations for scour depth accounting of densimetric Froude number, sediment size/sluice opening ratio and tailwater level/sluice opening ratio as independent parameters. To a greater degree, a sincere effort has been made by Aamir and Ahmad^16^ to evaluate the performance of ANN and ANFIS models to estimate maximum scour depth using an extensive experimental data set of attached wall jets. Chatterjee et al.^17^, Aderibigbe and Rajaratnam^18^, Dey and Sarkar^19^ and Aamir and Ahmad^8,20^ have also developed regression based equations for scour depth prediction. These studies only focussed on predicting scour depth, so a gap has been felt between scour depth and scour hole characteristics. Therefore, an attempt has been made to develop two multi-regression based models (linear and non-linear) and two machine learning technique based models for each scour hole parameters, i.e., depth of scouring, location of scour depth, height of the dune and location of dune crest. It is imperative to understand from Fig. 1 that ${d}_{s}$ is the maximum equilibrium scour depth (depth of scouring), ${x}_{s}$ is the distance to asymptotic depth of scour from the end of rigid apron (location of scour depth), ${d}_{d}$ is the height of dune and ${x}_{d}$ is the distance to maximum height of dune crest from the end of rigid apron (location of dune crest). Coming to AI based models, artificial neural network-particle swarm optimisation (ANN-PSO) and gene expression programming (GEP) are accounted in the study.

Further research on development of new methods for various parameters is always expected to understand a complete phenomenon. So, model development has been performed through multivariable regression analysis and two machine learning techniques, such as artificial neural network-particle swarm optimisation (ANN-PSO) and gene expression programming (GEP) using available data sets of Aamir and Ahmad^8^. The essential independent parameters considered in this study are densimetric Froude number, apron length, tailwater level, and median sediment size. Further, the comparison has been made in terms of different statistical parameters.

Figure 1 depicts a definition sketch of the scour hole developed under a wall jet. In this figure, ${d}_{s}$ = maximum equilibrium scour depth, ${x}_{s}$ = distance to asymptotic depth of scour from the end of rigid apron, ${x}_{o}$ = longitudinal extension of scour hole, ${d}_{d}$= height of dune, ${x}_{d}$ = distance to maximum height of dune crest from the end of rigid apron, $a$ = sluice opening, $V$ = issuing jet velocity, ${d}_{t}$ = tailwater depth, $L$ = length of the rigid apron. Maximum scour depth depends on various parameters, viz. sluice opening, sediment size, jet Froude number and tailwater depth. Since the jet is two dimensional, profile of the scour hole under wall jet is also two dimensional across the width of the flume. Therefore, the figure represents any longitudinal section of the profile of the scour hole.

Application of machine learning and soft computing techniques has been of interest to many researchers in prediction of scour depth under various hydraulic structures such as piers, abutments, spur dikes etc., but there has not been substantial work undertaken to develop soft computing techniques for prediction of scour depth and other scour hole characteristics under the influence of wall jets. The novelty of this paper lies in respect of using a wide range of data and predicting various scour characteristics, whereas the existing studies have only focused on the maximum scour depth. The developed soft computing models in the present study using such a wide range of data provide better prediction of scour characteristics for a wider range of parameters, which would facilitate better and more accurate prediction of scour characteristics for the reliable design of hydraulic structures. Additionally, the pivotal factors governing the scour characteristics have been identified using the Gamma test.

In the present study, four significant parameters i.e., depth of scouring, location of scour depth, height of the dune and location of dune crest are identified and also modelled to represent a complete phenomenon of scour hole formation caused by turbulent wall jets.

The present study aims to develop new independent mathematical relationships for these four parameters utilising the previous data sets. Multi Regression Analysis has been performed (linear and non-linear) to establish the relationships between the dependent parameters and influencing independent parameters. Further, artificial neural network-particle swam optimisation (ANN-PSO) and gene expression programming (GEP) based models are developed using the available data to compare the performance of all these models in predicting the four parameters. The intension of the present work is that if the parameters such as depth of scouring, location of scour depth, height of the dune and location of dune crest will be predicted, then one can visualise the total phenomenon. It is the extension of scour depth modelling because scour depth prediction only shows the depth in vertical axis. However, the present study tries to figure out whole phenomenon by predicting the horizontal and vertical dimensions of the whole phenomenon.

## Dimensional analysis

Dimensional analysis is used as a classical tool to identify the variables affecting the equilibrium scour depth. The functional form of equilibrium scour depth downstream of a stiff apron (smooth and rough) under submerged wall jets can be written as:

$$d_{s} \, = \,f(V,g,\nu ,\sigma_{g} ,\rho ,\rho_{s} ,a,L,d_{t} ,d_{50} ,k_{s} ),$$

where *ν* = kinematic viscosity of water; *σ*_*g*_ = geometric standard deviation of sediments; *ρ* = mass density of water; and *ρ*_*s*_ = mass density of sediments. For a two-phase flow phenomenon involving sediment–water interaction, the terms *g*, *ρ*, and *ρ*_*s*_ can appropriately be grouped as one independent parameter Δ*g* in functional representation of *d*_*s*_; where Δ = *s* − 1; *s* = relative density of sediments; *g* = acceleration due to gravity. Also, since the flow is turbulent, the effect of kinematic viscosity *ν* on maximum scour depth is negligible. *σ*_*g*_ also has a negligible effect upon maximum scour depth. Using the Buckingham π theorem, the following is obtained:

$$\frac{{d}_{s}}{a}=f\left(\frac{V}{\sqrt{\Delta g{d}_{50}}},\frac{L}{a}, \frac{{d}_{t}}{a}, \frac{{d}_{50}}{a}, \frac{{k}_{s}}{a}\right).$$

### Description of collected data

Chatterjee et al.^17^, Aderibigbe and Rajaratnam^18^ and Dey and Sarkar^19^ and Aamir and Ahmad^8^ indicated that, there are significant relationships existing between scouring depth and densimetric Froude number, apron length, tailwater level and median sediment size. They all have given the models for scour depth under wall jet. However, the data sets provided by previous researchers are not having the complete information about the distance to depth of maximum scour from the rigid apron $\left({x}_{0}\right)$, the height of dune $\left({d}_{d}\right)$ and the distance to maximum height of dune crest from the end of rigid apron $\left({x}_{d}\right)$. As Aamir and Ahmad^20^ have measured all these above parameters as given in Table 1, so their data sets are used in this study for modelling. All the four parameters are made non-dimensional by dividing them with sluice opening $\left(a\right)$. The study utilised a sample of 165 data points from the literature on turbulent wall jet phenomenon to establish the relationships among independent and dependent parameters. A range of different parameters of the used data is summarized in Table 1. In this table, F is issuing jet Froude number (= V/(g × d_50_)^0.5^) and* g* = acceleration due to gravity. The ratio of length of the rigid apron to sluice opening depth, i.e. L/a = 0, which signifies the absence of a rigid apron as the jet directly strikes the erodible bed as soon as it emerges from the sluice opening.

**Table 1:** Range of different parameters of the used data.

Investigator	Number of data	Range of input parameters				Output	Flume dimensions		
		*L/a*	*d*_*t*_*/a*	*D*_50_*/a*	*F*_*d*_	*d*_*s*_*/a*	Length (m)	Width (m)	Depth (m)
Aamir et al.^20^	167	20–100	6.667–20	0.018–0.534	4.28–40.34	0.333–20.2	10	0.6	0.54

## Methodology

This section discusses the comprehensive understanding of approaches considered in this study to develop the regression and machine learning based models for predicting the four parameters related to scour hole characteristics. Specifically, the purpose of the current study is to investigate the following four major things (a) what variables are predictive in the scouring phenomenon under turbulent wall jet, (b) How strong are independent variables at predicting different dependent parameters, (c) Developing regression based linear and nonlinear models and (d) compare the result with machine learning methods such as ANN-PSO and GEP. The answer to the first question is to model the maximum equilibrium scour depth $\left({d}_{s}\right)$, the distance to scour depth from the end of rigid apron $({x}_{0})$, the height of dune $\left({d}_{d}\right)$ and the distance to maximum height of dune crest from the end of rigid apron $\left({x}_{d}\right)$. To answer the second question, the Gamma test has been performed.

### Gamma test (GT)

Gamma Test is the first step to identify the best input combination. The base mean square error (MSE) that contributes to input data selections is measured by Gamma Test. The selected input data combination can be used as part of a non-linear model’s structure^21^. In this research, 15 different combinations of four input parameters (densimetric Froude number, apron length, tailwater level, and median sediment size) of each dependent parameter (i.e.,$\frac{{d}_{s}}{a}, \frac{{x}_{0}}{a}, \frac{{x}_{d}}{a} \text{and} \frac{{d}_{d}}{a}$) have been built in the winGamma software for wall jet scouring. Out of that, 7 different combinations including the best input combinations are presented for each dependent parameter in Tables 2, 3, 4 and 5. From Tables 2, 3, 4 and 5, it is observed that the combination of the four parameters with mask [1111] can establish better models for all four scouring hole parameters as compared to other combinations due to the lesser values of Gamma and V-ratio which are very close to zero.

**Table 2:** Selection of best input combination using Gamma test for $\frac{{d}_{s}}{a}$.

S. no.	Combination of input parameters	Γ	Std. error	V-ratio	Mask
1	*L/a, d*_*t*_*/a, D*_50_*/a, F*_*d*_	0.105867877	0.007740049	0.423471508	1111
2	*L/a, F*_*d*_	0.098069664	0.007201873	0.392278655	1001
3	*L/a, d*_*t*_*/a*	0.10147001	0.007135087	0.405880039	1100
4	*L/a, D*_50_*/a, F*_*d*_	0.095239933	0.006556656	0.38095973	1011
5	*L/a, d*_*t*_*/a, F*_*d*_	0.096638424	0.007637046	0.386553695	1101
6	*L/a, d*_*t*_*/a, D*_50_*/a*	0.093024543	0.005363532	0.372098171	1110
7	*d*_*t*_*/a, D*_50_*/a, F*_*d*_	0.101409335	0.008538391	0.405637339	0111

**Table 3:** Selection of best input combination using Gamma test for $\frac{{x}_{0}}{a}$.

S. no.	Combination of input parameters	Γ	Std. error	V-ratio	Mask
1	*L/a, d*_*t*_*/a, D*_50_*/a, F*_*d*_	0.08372749	0.010716791	0.334909962	1111
2	*L/a, F*_*d*_	0.0733234	0.009143858	0.293293599	1001
3	*L/a, d*_*t*_*/a*	0.080520733	0.010197675	0.322082934	1100
4	*L/a, D*_50_*/a, F*_*d*_	0.069894534	0.008164365	0.279578137	1011
5	*L/a, d*_*t*_*/a, F*_*d*_	0.072509357	0.006781383	0.290037429	1101
6	*L/a, d*_*t*_*/a, D*_50_*/a*	0.08096958	0.006843226	0.323878319	1110
7	*d*_*t*_*/a, D*_50_*/a, F*_*d*_	0.083049995	0.008666907	0.332199979	0111

**Table 4:** Selection of best input combination using Gamma test for $\frac{{x}_{d}}{a}$.

S. no.	Combination of input parameters	Γ	Std. error	V-ratio	Mask
1	*L/a, d*_*t*_*/a, D*_50_*/a, F*_*d*_	0.083407085	0.013707944	0.333628342	1111
2	*L/a, F*_*d*_	0.071059542	0.012013886	0.284238166	1001
3	*L/a, d*_*t*_*/a*	0.081122411	0.012951106	0.324489644	1100
4	*L/a, D*_50_*/a, F*_*d*_	0.068590864	0.01171195	0.274363456	1011
5	*L/a, d*_*t*_*/a, F*_*d*_	0.068313275	0.008672095	0.2732531	1101
6	*L/a, d*_*t*_*/a, D*_50_*/a*	0.081045038	0.009858291	0.324180153	1110
7	*d*_*t*_*/a, D*_50_*/a, F*_*d*_	0.081109677	0.008247759	0.32443871	0111

**Table 5:** Selection of best input combination using Gamma test for $\frac{{d}_{d}}{a}$.

S. no.	Combination of input parameters	Γ	Std. error	V-ratio	Mask
1	*L/a, d*_*t*_*/a, D*_50_*/a, F*_*d*_*,*	0.09647252	0.012018817	0.38589008	1111
2	*L/a, F*_*d*_	0.082396609	0.015646161	0.329586437	1001
3	*L/a, d*_*t*_*/a*	0.096979622	0.011195797	0.387918489	1100
4	*L/a, D*_50_*/a, F*_*d*_	0.083396426	0.017233225	0.333585705	1011
5	*L/a, d*_*t*_*/a, F*_*d*_	0.087962428	0.014333299	0.351849713	1101
6	*L/a, d*_*t*_*/a, D*_50_*/a*	0.108500501	0.013880282	0.434002004	1110
7	*d*_*t*_*/a, D*_50_*/a, F*_*d*_	0.097439784	0.011661279	0.389759135	0111

Another term, V-ratio is used to restore a scaled invariant clamour evaluated in the vicinity of 0 to 1 and can be used to arrange the GT performance and described as

1 $$V{\text{-}}ratio=\frac{\Gamma }{{\sigma }^{2}\left(y\right)},$$

where σ^2^(y) = variance of yield ‘y’, which provides a standardized measure of the Gamma statistic and allows a judgment to be formed independent of the yield range on the issue of how effectively the yield can be depicted by a smooth function^22^. The V-ratio is a measure for assessing the predictability of the given yields based on readily available data. It should be noted that the input combination with a low mean square error (MSE) and V-ratio value is regarded as the most suitable input combination for scour depth modeling. Before performing the analysis through all the soft computing techniques, the scour depths under wall jet are normalised (both the input and output data) to the domain [0.05, 0.95] using Eq. (6)^23^.

2 $${a}_{norm}=0.05+0.90\frac{\left(a-{a}_{min}\right)}{\left({a}_{max}-{a}_{min}\right)},$$

where *a*_*norm*_ = normalized input, *a* = original input, *a*_*min*_ = minimum of the input range, *a*_*max*_ = maximum of the input range.

To answer the third and fourth objectives, each of these four parameters is modelled through both linear and non-linear Multiple Regression analysis and compared with two AI based models i.e., ANN-PSO and GEP, in terms of statistical error analysis.

### Multiple regression analysis

Considering the significance of the criticality produced due to scour under wall jets, the present study aims to address this issue by developing two regression based models with available data as input parameters. Regression analysis is generally used to find the internal dependencies between a dependent parameter and one or more independent parameters^7,24^. The multiple regression model can be better than the uni-factorial or single regression model due to the consideration of more influencing parameters. The multiple regression analysis is an improvement upon the single regression analysis by analyzing several variables and deriving the relationships between a dependent variable and several independent variables. In this study, two Multi variable Regression models are considered such as multiple linear regression analysis and multiple non-linear regression analysis. Through, multiple linear regression (MLR), the resulted output function is a linear mathematical statement, represented as follows:

3 $${Y}_{1}={a}_{0}+{a}_{1}{X}_{1}+{a}_{2}{X}_{2}+\dots +{a}_{n}{X}_{n},$$

where ${Y}_{1}$ is the response variable, ${a}_{0}$–${a}_{n}$ are the constants of the equation, and ${X}_{1}$–${X}_{n}$ are the various independent variables. Multiple nonlinear regression (MNLR) is an illustration of regression analysis in which nonlinear combinations of both the input and output parameters are analysed. MLR constitutes the linear models and MNLR can establish models of nonlinear relationships between influencing and response variables^7^. The MNLR is represented as follows:

4 $${Y}_{1}={b}_{0}{{X}_{1}}^{{b}_{1}}.{b}_{2}{{X}_{2}}^{{b}_{2}}\dots {b}_{n}{{X}_{n}}^{{b}_{n}},$$

where ${b}_{0}$–${b}_{n}$ are the equation parameters.

### Artificial neural network-particle swarm optimization (ANN-PSO)

The soft computing tools in predicting the scour depth have caught much attention due to their simplicity and accuracy in computation. In contrast with various hydraulic structures such as piers, abutments, and spur dikes, it is found that there is a limited work conducted specifically for scour hole characteristics for wall jets problem. Here, both ANN-PSO and GEP are applied to the computation of all the four parameters such as depth of scouring, location of scour depth, depth of the dune and location of dune crest. The process of setting parameters for ANN-PSO (Artificial Neural Network-Particle Swarm Optimization) and GEP (Gene Expression Programming) involves careful consideration to ensure optimal performance and reliability. ANN-PSO, also known as Artificial Neural Network-Particle Swarm Optimization, is a hybrid computational technique that combines the capabilities of Artificial Neural Networks (ANN) and Particle Swarm Optimization (PSO) algorithms. This approach is extensively employed in various fields, such as in optimization, pattern recognition, data mining, and machine learning. The hybrid ANN-PSO approach aims to hold the strengths of both ANN and PSO to intensify the optimization process and enhance the performance of neural networks. The PSO algorithm assists to find superior sets of weights for the neural network, resulting greater accuracy and faster convergence during training. This is done by searching the efficient parameter space, exploring and analysing different weight combinations, and adjusting the ANN’s parameters to find an optimal solution for the specified problem.

**Figure 2:**
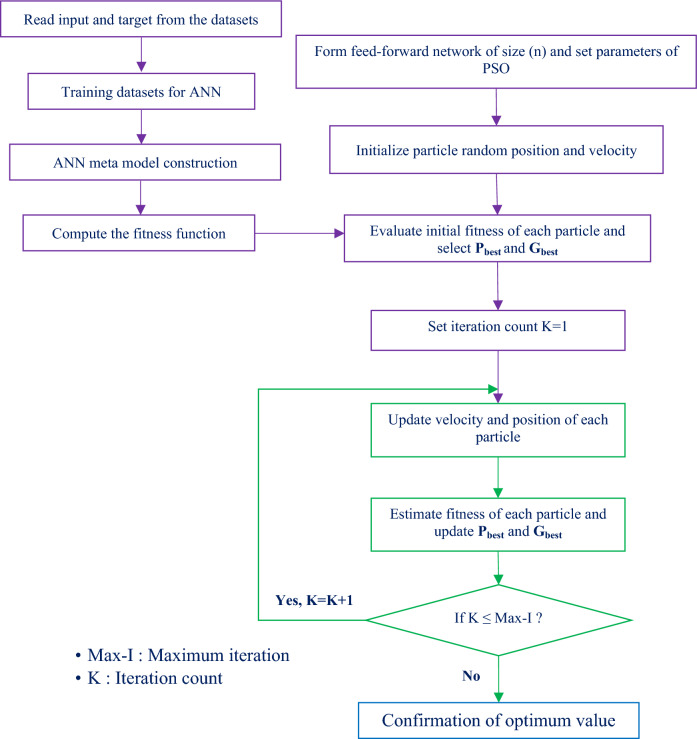
Flow chart for the methodology of the ANN–PSO algorithm.

The hybrid ANN-PSO model is widely used in many different fields since it can attain higher accuracy in less time. The ANN-PSO model approach starts with the initialization of a set of random particles. The population of particles is known as a swarm. This step specifies the positions of particles that reflect the ANN connection factors, such as biases and weights. Particle selection is normally done at random. Starting with a random population of solutions, the system iteratively improves these solutions in an attempt to find the best solution within a given search space. The hybrid PSO model network is then trained using the particles’ initial positions (along with their initial biases and weights). The fitness of the trained model can be calculated using the difference between the actual and observed output. With each iteration, the solution guides the swarm toward the optimal goal by employing each particle’s ability to rely on the expertise of others. Each subsequent iteration characterizes two values, local best (*p*_*id*_) and global best (*p*_*gd*_)^25,26^. The ‘global best’ is the best position among all previously obtained individual best positions, whereas the ‘local best’ is the best position attained by a particle so far. Weights are introduced (Mohandes^27^), allowing particles to achieve balance throughout global and local exploration.

5 $${{v}_{id}}^{new}=Wi. {{v}_{id}}^{old}+{C}_{1}.{R}_{1}.\left({p}_{id}-{x}_{id}\right)+{C}_{2}.{R}_{2}.\left({p}_{gd}-{x}_{id}\right),$$

6 $${{x}_{id}}^{new}={{v}_{id}}^{new}+{{x}_{id}}^{old},$$

where *R*_*1*_ and *R*_*2*_ are random values ranging from zero to unity, *C*_1_ and *C*_2_ are acceleration constants that typically range from 1 and 3, *p*_*id*_ and *p*_*gd*_ represent the individual and global best values, and *W*_1_ is the inertia weight. The acceleration coefficients *C*_1_ and *C*_2_, which represent the cognitive and social learning factors, respectively, have an impact on both the local and global optimal solutions during the optimization process. *C*_1_ and *C*_2_ represent the weights assigned to the top historical position and the highest global position, respectively. For ANN-PSO, key parameters such as inertia weight, cognitive and social coefficients, and swarm size significantly impact the convergence and stability of the optimization process. Literature such as Shi and Eberhart^28,29^, Clerc and Kennedy^30^, and Chatterjee and Siarry^31^ provide foundational insights into these parameter settings, emphasizing empirical adjustments and nonlinear variations for dynamic adaptation. In contrast, the global best signifies the best position attained among all individual best positions up to that point. To strike a balance between the global and local exploration capabilities of the particles, an inertia weight (*w*) is employed. Additionally, *C*_1_ and *C*_2_ represent the individual and social learning rates, typically varying between 1 and 3 with an interval of 0.25^32,33^. Genetic algorithms (GA) and particle swarm optimization (PSO) use evolutionary systems to find solutions. To optimize the best model, the input parameters may vary, such as the number of neurons (N) between 5 and 10 and the swarm population size between 10 and 200 with an iteration value of 1000^34^. The flow chart for the methodology of ANN-PSO model is depicted in Fig. 2.

#### Gene expression programming (GEP)

GEP, is an extension of genetic programming which evolves computer programs such as different mathematical expressions, decision trees, polynomial constructs, and logical expressions. Computer programs generated by GEP are encoded in linear chromosomes and are further interpreted into expression trees (ETs) (Ferreira 2001). GEP is a full-fledged genotype/phenotype system, where the genotype is completely separated from the phenotype. The approach proposes to employ the GEP design to form nonlinear functions to analyze nonlinear parameters^35,36^. The GEP structure can be used as a guide to arrange the values of the mutation, inversion, one-, two-, and gene recombination rates in order to accommodate different mathematical operators and appropriately produce the desired outcome^22,37,38^. For GEP and genetic algorithms, the selection of genetic operators like mutation rates, crossover rates, and population size is crucial. Foundational works by Goldberg^39^, Srinivas and Patnaik^40^, and Ferreira^41^ presented the effects of these parameters on genetic diversity and convergence. Adaptive strategies for parameter tuning, as discussed by Eiben and Smit^42^, further enhance algorithm performance by adjusting parameters in response to the evolving population dynamics. Several of these genetic operators used for chromosome genetic alteration were explained in the GEP scheme^43^. The modelling includes five major steps to prepare to use GEP. The first is to choose the fitness function. For this problem, the fitness,* f*_*i*_, of an individual program, *i*, is measured by:

7 $${f}_{i}=\sum_{j=1}^{{C}_{t}}\left(M-\left|{C}_{\left(i,j\right)}-{T}_{j}\right|\right),$$

where *M* is the range of selection, *C*_(*i*,*j*)_ is the value returned by the individual chromosome *i* for fitness case *j* (out of *C*_*t*_ fitness cases), and *T*_*j*_ is the target value for fitness case *j*. The advantage of this type of fitness function is that it allows the system to find the optimal solution autonomously.

Second, the set of terminals *T* and the set of functions *F* are chosen to create the chromosomes. In this study, for four equations developed to predict *d*_*s*_/*a*, *x*_0_/*a*, *x*_*d*_/*a* and *d*_*d*_/*a*, the terminals include four independent parameters (*L/a*, *d*_*t*_*/a*, *D*_50_*/a*, *F*_*d*_). These parameters are derived from the optimal input combination obtained from Gamma test (Tables 2, 3, 4, 5). Chromosomes represent complete solutions. A common length for chromosomes is 30–50 genes, depending on the complexity of the problem. In this study, length for chromosomes is considered 28 and 30. To determine the appropriate function set, it is essential to review previous investigations in this area. Consequently, four basic operators (+, −, *, /) and fundamental mathematical functions (power, exponential, *ln*) were applied for modelling.

The third major step is to choose the chromosomal architecture, specifically the length of the head and the number of genes. Initially, a single gene and two head lengths were used, with the number of genes and head lengths incrementally increased one at a time during each run while monitoring the training and testing performances of each case. It was observed that using more than two genes and a head length greater than 8 did not significantly improve the training and testing performance of the GEP models. Therefore, a head length of 8 and three genes per chromosome are employed for each GEP model in this study.

The fourth step is to choose the linking function. In this study, addition operator are used as linking functions. The fifth and final step is to select the set of genetic operators that induce variation and their rates. A combination of all genetic operators (mutation, inversion, and crossover) is used for this purpose. Mutation rate, the probability of altering a gene, is typically set between 0.01 and 0.1. This rate introduces diversity while maintaining stability. Inversion rate, the probability of reversing a segment within a chromosome, is often set between 0.01 and 0.1. This helps preserve genetic material while creating new sequences. Crossover frequency, the likelihood of parent chromosomes exchanging segments, is generally high, around 0.3 to 0.9. This ensures significant genetic mixing. In the present study, mutation rate, inversion rate and crossover frequency are taken as 0.044, 0.1 and 0.5 respectively.

**Figure 3:**
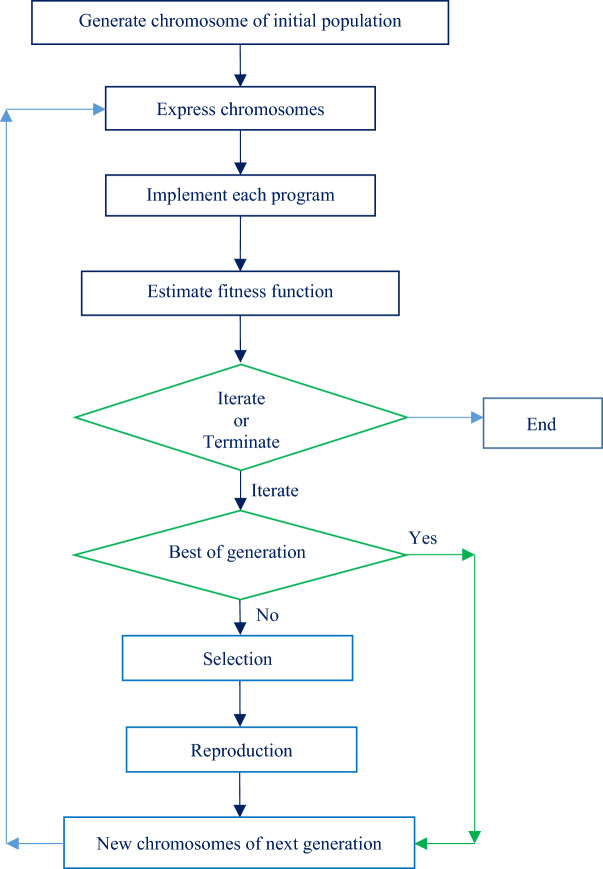
Brief algorithm of gene expression programming (GEP) model.

The present work utilises the GEP to estimate the four scour hole parameters by adopting an innovative architecture of GEP structure. The flow chart of the methodology of GEP model is depicted in Fig. 3.

## Uncertainty and reliability analysis

The importance of uncertainty and reliability analyses in evaluating model performance is crucial for ensuring the credibility and utility of predictive models^44–48^. Uncertainty analysis aims to define a reliable uncertainty interval, denoted as U95, which indicates the range within which the true outcome of an experiment is likely to fall. U95 is estimated based on the errors in the experimental measurement process, with the understanding that in approximately 95 out of 100 trials, the true outcome will lie within this interval. The U95 formula involves the weighted summation of squared differences between observed and predicted values. Reliability analysis evaluates a model’s overall consistency, expressed as a percentage calculated through the relative average error (RAE). The reliability factor is set to 1 if the RAE is less than or equal to a threshold (typically 20%), and the model’s reliability is determined as the average of these factors. Collectively, these analyses provide a comprehensive understanding of model behavior, enabling decision-makers to make more informed choices and enhancing the overall robustness and trustworthiness of predictive models across various domains.

## Results and discussion

**Figure 4:**
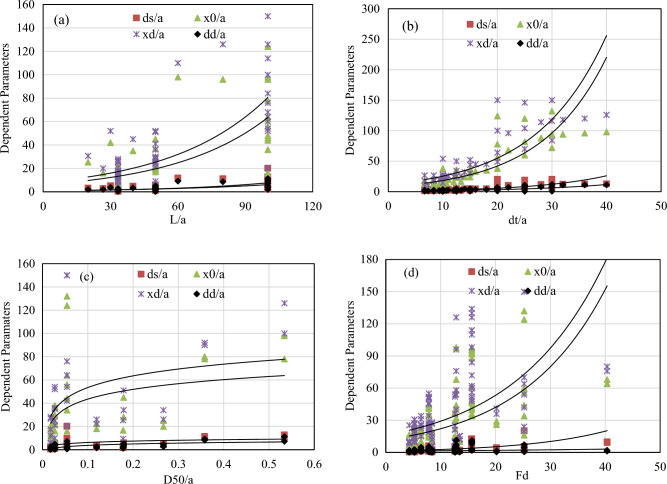
Relationship of independent and dependent parameters (d_s_/a, × 0/a, xd/a, dd/a) (**a**) L/a, (**b**) d_t_/a, (**c**) D_50_/a, (**d**) F_d_.

Considering all the necessary data for the analysis, both multiple linear regression analysis (MLRA) and non-linear multiple regression analysis (MLRA) are performed. The nondimensional dependent variables considered in this study are maximum equilibrium scour depth $\left({d}_{s}/a\right)$, the distance to scour depth from the end of rigid apron $\left({x}_{0}/a\right)$, the height of dune $\left({d}_{d}/a\right)$ and the distance to maximum height of dune crest from the end of rigid apron $\left({x}_{d}/a\right)$. From Gamma test, densimetric Froude number (F_d_), apron length (L), tail water level (d_t_), median sediment size (D_50_) are found to be influencing these four dependent parameters. For modelling, apron length (L), tail water level (d_t_) and median sediment size (D_50_) are made dimensionless numbers by dividing them with height of gate opening (a) such as L/a, d_t_/a, D_50_/a respectively. The results of variation of dependent parameters with input parameters are analyzed as demonstrated in Fig. 4a–d. Present research reports a rising trend between all dependent parameters against the apron length (L/a), as shown in Fig. 4a. The reason for this trend is attributable to the dissipation of energy of the jet as it travels over the apron. Hence, longer aprons are able to dissipate larger energy and reduce the erosive capacity of the jet. Similarly, rising trends are also visible for the variations of dependent parameters with tail water level (D_t_/a), densimetric Froude number (F_d_), and median sediment size (D_50_), as shown in Fig. 4.

A number of single regressions models for maximum equilibrium scour depth $\left({d}_{s}/a\right)$, the distance to scour depth from the end of rigid apron $\left({x}_{0}/a\right)$, the height of dune $\left({d}_{d}/a\right)$ and the distance to maximum height of dune crest from the end of rigid apron $\left({x}_{d}/a\right)$ with different input parameters are established. After rigorous study, the best models for each couple (dependent vs independent) with high coefficient of determination R^2^ are identified. Then, multiple regression analysis has been performed and two equations (one for linear and another for nonlinear cases) are obtained for each dependent parameter, as provided below.

### Resulted equations from multiple linear regression analysis (MLRA)

MLRA for maximum equilibrium scour depth $\left({d}_{s}/a\right)$

8 $$\frac{{d}_{s}}{a}=0.293\left({d}_{t}/a\right)-0.055\left(L/a\right)+7.489\left({d}_{50}/a\right)+0.270{F}_{d}-1.437,$$

MLRA for distance to scour depth from the end of rigid apron $\left({x}_{0}/a\right)$

9 $$\frac{{x}_{0}}{a}=2.295\left({d}_{t}/a\right)-0.371\left(L/a\right)+58.996\left({d}_{50}/a\right)+1.807{F}_{d}-12.317,$$

MLRA for distance to maximum height of dune crest from the end of rigid apron $\left({x}_{d}/a\right)$

10 $$\frac{{x}_{d}}{a}=2.61\left({d}_{t}/a\right)-0.433\left(L/a\right)+83.677\left({d}_{50}/a\right)+2.204{F}_{d}-13.661.$$

MLRA for the height of dune $\left({d}_{d}/a\right)$.

11 $$\frac{{d}_{d}}{a}=0.279\left({d}_{t}/a\right)-0.062\left(L/a\right)+1.476\left({d}_{50}/a\right)+0.071{F}_{d}-0.573.$$

### Resulted equations from multiple non-linear regression analysis (MNLRA)

MNLRA for maximum equilibrium scour depth $\left({d}_{s}/a\right)$

12 $$\frac{{d}_{s}}{a}=6.395 {\left(\frac{L}{a}\right)}^{-1.378}{\left(\frac{{d}_{t}}{a}\right)}^{0.90}{\left(\frac{{D}_{50}}{a}\right)}^{0.465}{\left({F}_{d}\right)}^{1.469}.$$

MNLRA for distance to scour depth of from the end of rigid apron $\left({x}_{0}/a\right)$

13 $$\frac{{x}_{0}}{a}=36.03 {\left(\frac{L}{a}\right)}^{-1.199}{\left(\frac{{d}_{t}}{a}\right)}^{0.828}{\left(\frac{{D}_{50}}{a}\right)}^{0.473}{\left({F}_{d}\right)}^{1.393}.$$

MNLRA for the distance to maximum height of dune crest from the end of rigid apron $\left({x}_{d}/a\right)$

14 $$\frac{{x}_{d}}{a}=40.94 {\left(\frac{L}{a}\right)}^{-1.04}{\left(\frac{{d}_{t}}{a}\right)}^{0.753}{\left(\frac{{D}_{50}}{a}\right)}^{0.466}{\left({F}_{d}\right)}^{1.258}.$$

MNLRA for the height of dune $\left({d}_{d}/a\right)$

15 $$\frac{{d}_{d}}{a}=18.395 {\left(\frac{L}{a}\right)}^{-1.534}{\left(\frac{{d}_{t}}{a}\right)}^{1.08}{\left(\frac{{D}_{50}}{a}\right)}^{0.703}{\left({F}_{d}\right)}^{1.022}.$$

The coefficient of determination R^2^ for multiple linear regression equation and multiple nonlinear regression equation are found to be 0.56 for $\frac{{d}_{s}}{a}$, 0.66 for $\frac{{x}_{0}}{a}$, 0.67 for $\frac{{x}_{d}}{a}$, 0.56 for $\frac{{d}_{d}}{a}$ and 0.66 for $\frac{{d}_{s}}{a}$, 0.74 for $\frac{{x}_{0}}{a}$, 0.74 for $\frac{{x}_{d}}{a}$, 0.54 for $\frac{{d}_{d}}{a}$ respectively which measures the percentage of how much of the total variance is explained by the independent variables. Further, an attempt has been made to apply two machine learning approaches such as ANN-PSO and GEP to model these four parameters ${d}_{s}/a$, ${x}_{0}/a$, ${d}_{d}/a$ and ${x}_{d}/a$.

#### Model development using ANN-PSO

In this ANN-PSO modelling, several trials were performed and the coefficients C_1_ and C_2_ were fixed at 1 and 2.5, 2 and 2.5, 1.5 and 2.5, 1.5 and 2.5 for ${d}_{s}/a$, ${x}_{0}/a$, ${d}_{d}/a$ and ${x}_{d}/a$ respectively. The error analysis results for the training data, testing data, and the entire dataset for various swarm sizes and number of neurons (N) for each dependent parameter were analysed. It was observed that the swarm size increases with the same values of C_1_ and C_2_. While maintaining the number of neurons constant, the values of R^2^, E, and I_d_ decrease, while the value of RMSE increases.

#### Model development using GEP

In this section, model development for four dependent parameters using the GEP approach is described. By incorporating all the four independent input parameters (L/a, d_t_/a, D_50_/a, F_d_), GEP expression has been derived and GeneXpro Tools 5.0 software package is used for this analysis. Using normalized data, four attempts have been made with the variation of chromosome number, fitness function, and number of runs for modelling the wall jet scouring. Table 6 shows the corresponding parameters of the optimized GEP model.

**Figure 5:**
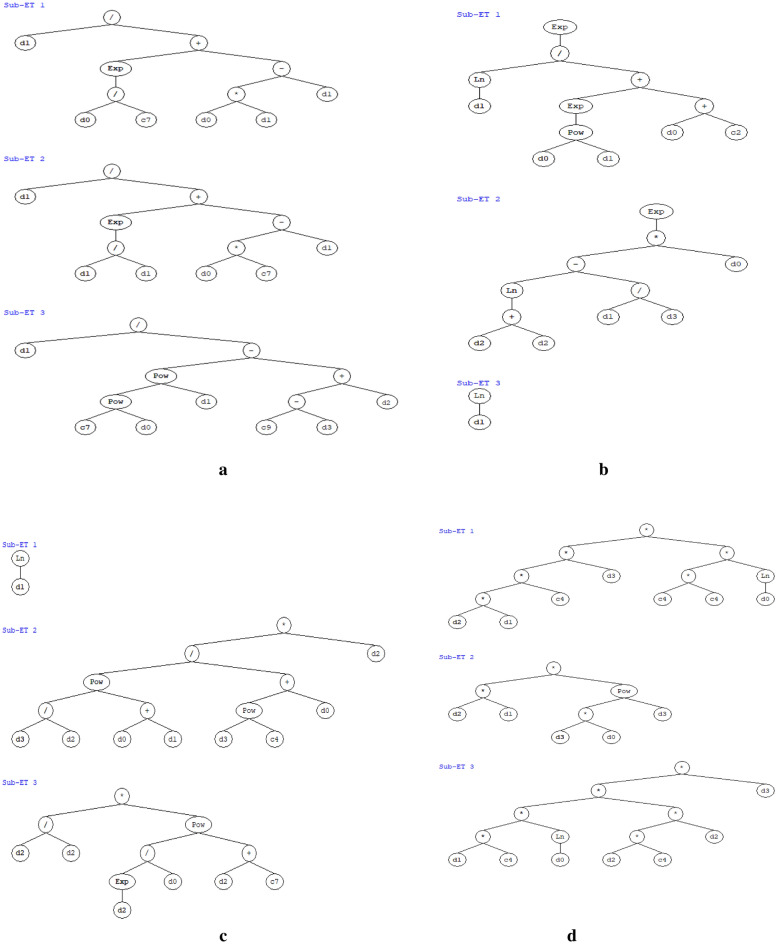
(**a**) Expression tree for Wall jet scouring of ${d}_{s}/a$. (**b**) Expression tree for Wall jet scouring of ${x}_{0}/a$. (**c**) Expression tree for Wall jet scouring of ${x}_{d}/a$. (**d**) Expression tree for Wall jet scouring of ${d}_{d}/a$.

The expression trees for models of ${d}_{s}/a$, ${x}_{0}/a$, ${d}_{d}/a$ and ${x}_{d}/a$ are presented in Fig. 5a–d, respectively. In this expression tree, *d*_0_ = *L/a*, *d*_1_ = *d*_*t*_*/a, d*_2_ = *D*_50_/*a* and *d*_3_ = *F*_*d*_. In Sub-ET 1, 2 and 3 (Fig. 5a), C_7_ and C_9_ are constants, and their values are 3.45 and − 5.56 respectively for model of ${d}_{s}/a$. In Sub-ET 1 (Fig. 5b), C_2_ is constant, the value is − 8.93 for model of ${x}_{0}/a$. In Sub-ET 2 and 3 (Fig. 5c), C_4_ and C_7_ are the constants, and their values are 2.971 and − 0.376 respectively for model of ${x}_{d}/a$. In Sub-ET 1 and 3 (Fig. 5d), C_4_ is constant, the value is − 3.114 and 3.145 respectively for model of ${d}_{d}/a$. The equations derived from the expression trees are presented in Eqs. (16)–(19).

**Table 6:** Parameters of the optimized GEP model for Wall Jet scouring.

Serial no.	Parameter	Parameter setting			
		For ${d}_{s}/a$	For ${x}_{0}/a$	For ${x}_{d}/a$	For ${d}_{d}/a$
1	Chromosomes	28	30	30	30
2	Genes	3	3	3	3
3	Head size	8	8	8	8
4	Number of generation	15,774	15,367	15,243	15,639
5	Mutation rate	0.044	0.044	0.044	0.044
6	Inversion rate	0.1	0.1	0.1	0.1
7	Cross-over frequency	0.5	0.5	0.5	0.5
8	Two-point recombination rate	0.3	0.3	0.3	0.3
9	One point recombination rate	0.3	0.3	0.3	0.3
10	Gene transposition rate	0.1	0.1	0.1	0.1
11	Gene recombination rate	0.1	0.1	0.1	0.1
12	Linking function	Addition	Addition	Addition	Addition
13	Function set	+, −, *, /, power, exponential, ln	+, −, *, /, power, exponential, ln	+, −, *, /, power, exponential, ln	+, −, *, /, power, exponential, ln
14	Program size	26	24	29	30

Expression for $${d}_{s}/a$$:

16 $$\frac{{d}_{s}}{a}=\left[\frac{\left(\frac{{d}_{t}}{a}\right)}{\left({e}^{\frac{(L/a)}{3.45}}\right)+\left[\left(\frac{L}{a}\times \frac{{d}_{t}}{a}\right)-\frac{{d}_{t}}{a}\right]}\right]+\left[\frac{\left(\frac{{d}_{t}}{a}\right)}{2.187+\left[\left(\frac{L}{a}\times 3.45\right)-\frac{{d}_{t}}{a}\right]}\right]+\left[\frac{\left(\frac{{d}_{t}}{a}\right)}{{\left({3.45}^\frac{L}{a}\right)}^{\frac{{d}_{t}}{a}}+\left[\left(5.56+{F}_{d}\right)-\left(\frac{{D}_{50}}{a}\right)\right]}\right].$$

Expression for $${x}_{0}/a$$:

17 $$\frac{{x}_{0}}{a}={e}^{\left(\frac{\text{ln}\left(\frac{{d}_{t}}{a}\right)}{{e}^{\frac{L}{a}^{{d}_{t}/a}}+\left(\frac{L}{a}-8.93\right)}\right)}+{e}^{\left(\left({\text{ln}}\left(2\times \frac{{D}_{50}}{a}\right)-\frac{\left(\frac{{d}_{t}}{a}\right)}{{F}_{d}}\right)\times \frac{L}{a}\right)}+{\text{ln}}\left(\frac{{d}_{t}}{a}\right).$$

Expression for $${x}_{d}/a$$:

18 $$\frac{{x}_{d}}{a}={\text{ln}}\left(\frac{{d}_{t}}{a}\right)+\left[\left(\frac{{\left(\frac{{F}_{d}}{\left(\frac{{D}_{50}}{a}\right)}\right)}^{\left(\frac{L}{a}+\frac{{d}_{t}}{a}\right)}}{{{F}_{d}}^{2.971}+\frac{L}{a}}\right)\times \left(\frac{{D}_{50}}{a}\right)\right]+\left[1\times {\frac{{e}^{\frac{{D}_{50}}{a}}}{\left(\frac{L}{a}\right)}}^{\left(\frac{{D}_{50}}{a}-0.376\right)}\right].$$

Expression for $${d}_{d}/a$$:

19 $$\frac{{d}_{d}}{a}=\left[\left(\frac{{D}_{50}}{a}\times \frac{{d}_{t}}{a}\right)\times \left(-3.114\times {F}_{d}\right)\times 9.697\times {\text{ln}}\left(\frac{L}{a}\right)\right]+\left(\frac{{D}_{50}}{a}\times \frac{{d}_{t}}{a}\right)\times {\left({F}_{d}\times \frac{L}{a}\right)}^{{F}_{d}}+\left[\left(\left(\left(\frac{{d}_{t}}{a}\times 3.145\right)\times {\text{ln}}\left(\frac{L}{a}\right)\right)\times {\left(\frac{{D}_{50}}{a}\right)}^{2}\times 3.145\right)\times {F}_{d}\right].$$

**Figure 6:**
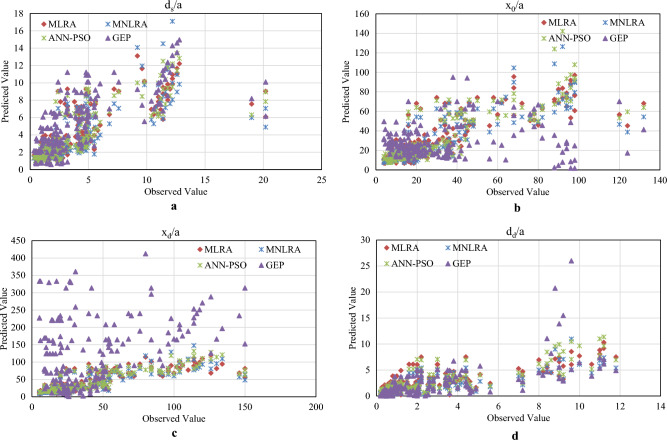
(**a**) Observed vs predicted value for all the model of ${d}_{s}/a$. (**b**) Observed vs predicted value for all the model of ${x}_{0}/a$. (**c**) Observed vs predicted value for all the model of ${x}_{d}/a$. (**d**) Observed vs predicted value for all the model of ${d}_{d}/a$.

Figure 6a–d shows the relationship between observed and predicted values for the model of ${d}_{s}/a$, ${x}_{0}/a$, ${x}_{d}/a$ and ${d}_{d}/a$ respectively. It is observed that the predicted model of ANN-PSO gives good agreement with observed values for all the four models, whereas GEP shows the unsatisfactory result of the present study.

#### Performance of uncertainty and reliability analysis

To perform a comprehensive statistical assessment of the proposed models, two indices namely confidence interval (U95) and reliability index are computed. The statistical evaluation of the present models, highlighting their predictive capabilities and robustness using uncertainty analysis and reliability index, is presented in Table 7.

**Table 7:** Comparison of performance results for the uncertainty and reliability analysis.

Models	For $\frac{{d}_{s}}{a}$		For $\frac{{x}_{0}}{a}$		For $\frac{{x}_{d}}{a}$		For $\frac{{d}_{d}}{a}$	
	Confidence interval (U95)	Reliability index	Confidence interval (U95)	Reliability index	Confidence interval (U95)	Reliability index	Confidence interval (U95)	Reliability index
MLRA	0.402	0.461	2.604	0.467	3.101	0.479	0.293	0.448
MNLRA	0.415	0.521	2.598	0.545	3.063	0.570	0.301	0.497
ANN-PSO	**0.383**	**0.573**	**2.539**	**0.591**	**2.805**	**0.576**	**0.268**	**0.548**
GEP	0.483	0.291	28.800	0.206	19.276	0.170	0.409	0.40

The best results are shown in bold.

Table 7 shows the confidence interval (U95) and reliability index (RI) of MLRA, MNLRA, ANN-PSO and GEP in predicting ${d}_{s}/a$, ${x}_{0}/a$, ${x}_{d}/a$ and ${d}_{d}/a$. ANN-PSO model represented the lowest values of confidence interval (U95), i.e., 0.383, 2.539, 2.805 and 0.268 when compared to MLRA (0.402, 2.604, 3.101and 0.293), MNLRA (0.415, 2.598, 3.063 and 0.301) and GEP (0.483, 28.800, 19.276 and 0.409) for predicting ${d}_{s}/a$, ${x}_{0}/a$, ${x}_{d}/a$ and ${d}_{d}/a$ respectively. Additionally, predictions of ${d}_{s}/a$, ${x}_{0}/a$, ${x}_{d}/a$ and ${d}_{d}/a$ provided by ANN-PSO are more reliable (RI = 0.573, 0.591, 0.576 and 0.548) when compared to other present models. Moreover, MNLRA shows slightly less reliable (RI = 0.521, 0.545, 0.570 and 0.497) than ANN-PSO in predicting ${d}_{s}/a$, ${x}_{0}/a$, ${x}_{d}/a$ and ${d}_{d}/a$. GEP shows wider confidence intervals (U95) and lower relative index, indicating higher uncertainty and less reliable model in predicting ${d}_{s}/a$, ${x}_{0}/a$, ${x}_{d}/a$ and ${d}_{d}/a$. This analysis suggests that the ANN-PSO provides a more consistent and reliable model for the prediction of ${d}_{s}/a$, ${x}_{0}/a$, ${x}_{d}/a$ and ${d}_{d}/a$.

#### Statistical error analysis

This section illustrates the performance of the two soft-computing models and two multiple regression models in predicting ${d}_{s}/a$, ${x}_{0}/a$, ${x}_{d}/a$ and ${d}_{d}/a$. To assess the strength of present approaches, seven statistical indices are accounted including two statistical indices such as Root mean square error (RMSE) and coefficient of determination (R^2^), and two relative indices, E and I_d_^38,49–52^. The error indices are computed for all the present models in terms of MAE, MAPE, MSE, RMSE, R^2^, E and I_d_ are depicted in Table 8.

From Table 8, it is found that for both $\left({d}_{s}/a\right)$ and $\left({x}_{0}/a\right)$, the error indices, i.e., MAE, MAPE, MSE and RMSE are less for MLRA and MNLRA as compared to the ANN-PSO and GEP. But, the error indices, i.e., MAE, MAPE, MSE and RMSE are found to be less for ANN-PSO as compared to MLRA, MNLRA and GEP for both ${x}_{d}/a$ and ${d}_{d}/a$. However, the R^2^ value is more in ANN-PSO model for all predicting parameter values. E and Id values are also found to be close to 1 for ANN-PSO models for all three predicting parameter values except $\left({x}_{0}/a\right)$. For $\left({x}_{0}/a\right)$, E and Id values are found to be close to 1 for MLRA model. By comparing all the statistical parameters, ANN-PSO model shows better result as compared to the other presented regression and soft computing techniques (Table 8).

**Table 8:** Error analysis of different approaches in estimating ${d}_{s}/a$, ${x}_{0}/a$, ${d}_{d}/a$ and ${x}_{d}/a$ for wall jet scouring.

	Method	MAE	MAPE	MSE	RMSE	R^2^	E	Id
${d}_{s}/a$	MLRA	1.773	48.752	6.927	2.632	0.565	0.565	0.776
	MNLRA	1.704	37.015	7.383	2.717	0.549	0.536	0.752
	ANN-PSO	1.585	39.962	6.309	1.512	0.605	0.604	0.805
	GEP	2.346	94.267	10.016	3.165	0.483	0.371	0.758
${x}_{0}/a$	MLRA	11.578	47.666	6.927	12.632	0.565	0.992	0.996
	MNLRA	11.139	38.276	289.886	17.026	0.665	0.657	0.833
	ANN-PSO	11.011	44.250	277.028	6.644	0.681	0.672	0.865
	GEP	154.3884	128.30	35,626.21	188.749	0.542	− 41.180	0.009
${x}_{d}/a$	MLRA	13.526	43.564	412.946	20.321	0.667	0.667	0.840
	MNLRA	13.109	36.012	403.071	20.077	0.682	0.675	0.841
	ANN-PSO	12.008	29.929	338.046	6.386	0.727	0.727	0.879
	GEP	93.638	85.285	15,958.73	126.328	0.113	− 11.872	0.355
${d}_{d}/a$	MLRA	1.433	52.761	358.651	18.938	0.562	− 41.602	− 19.549
	MNLRA	1.378	45.588	3.887	1.971	0.591	0.538	0.714
	ANN-PSO	1.272	24.855	3.076	1.754	0.636	0.635	0.846
	GEP	1.667	67.576	7.177	2.679	0.427	0.148	0.703

## Conclusions

The present study has focused on modeling of four geometrical variables which represent the complete scour hole formation based on experimental data sets. Total 167 data points have been utilised for the modelling of following parameters, such as the maximum equilibrium scour depth $\left({d}_{s}/a\right)$, the distance to maximum scour depth from the end of rigid apron $\left({x}_{0}/a\right)$, the height of dune $\left(d/a\right)$ and the distance to maximum height of dune crest from the end of rigid apron $\left({x}_{d}/a\right)$ render a complete scour hole phenomenon. The gamma test reveals that densimetric Froude number, apron length, tail water level, and median sediment size, are the most critical influencing parameters to predict the four significant parameters, associated with scour depth characteristics. In this study, for the prediction of scouring depths, two multiple (linear and non-linear) regression models and two soft computing techniques such as artificial neural network-particle swarm optimisation (ANN-PSO) and gene expression programming (GEP) models are employed. Particle Swarm Optimization (PSO) enhances model performance by reducing uncertainty and improving reliability. ANN-PSO demonstrates the most efficient and robust models through comprehensive uncertainty and reliability analyses.

To find the best performer among four models in predicting the dependent parameters, seven statistical indices are used. For ${d}_{s}/a$, ANN-PSO gives better result with maximum R^2^, E and Id values and minimum RMSE, MAE, MAPE and MSE values as compared to the other models that are MLRA, and MNLRA and GEP. However, for ${x}_{0}/a$, the results shown by MLRA are found to be better than the other models. Further, the ANN-PSO gives less errors for predicting both ${x}_{d}/a$ and ${d}_{d}/a$ with high R^2^, E and Id values of 0.727, 0.727, 0.879 and 0.636, 0.635, 0.846 respectively. Overall, by analysing all the statistical parameters, ANN-PSO model shows good accuracy and predicts well as compared to other presented models.

More experimental investigations need to be conducted as there are very few data sets are available for both input and out parameters. The limitation of the present approach is the range of data sets used in the modelling of the parameters associated with scour depth formation. Some newer soft computing techniques such as GMDH, SVM and M5 Tree model may be applied and compared in future work to provide better results if the input parameter values lie in the same range as accounted during its development. As this phenomenon is very dynamic, this research can further be improved considering wider ranges of data sets.

## Data Availability

All the data used in this study is available from the corresponding author upon request.
